# *Lactococcus lactis* carrying the pValac DNA expression vector coding for IL-10 reduces inflammation in a murine model of experimental colitis

**DOI:** 10.1186/1472-6750-14-73

**Published:** 2014-08-09

**Authors:** Meritxell Zurita-Turk, Silvina del Carmen, Ana CG Santos, Vanessa B Pereira, Denise C Cara, Sophie Y Leclercq, Alejandra dM de LeBlanc, Vasco Azevedo, Jean-Marc Chatel, Jean G LeBlanc, Anderson Miyoshi

**Affiliations:** 1 Department of General Biology, Institute of Biological Sciences, Federal University of Minas Gerais, Belo Horizonte, Brazil; 2 Reference centre for Lactobacilli (CERELA-CONICET), San Miguel de Tucumán, Argentina; 3 Department of Biochemistry and Immunology, Institute of Biological Sciences, Federal University of Minas Gerais, Belo Horizonte, Brazil; 4 Department of Morphology, Institute of Biological Sciences, Federal University of Minas Gerais, Belo Horizonte, Brazil; 5 Ezequiel Dias Foundation (FUNED), Belo Horizonte, Brazil; 6 INRA, UMR1319 Micalis, Domaine de Vilvert, F-78350 Jouy-en-Josas, France

## Abstract

**Background:**

Inflammatory bowel diseases (IBD) are intestinal disorders characterized by inflammation in the gastrointestinal tract. Interleukin-10 is one of the most important anti-inflammatory cytokines involved in the intestinal immune system and because of its role in downregulating inflammatory cascades, its potential for IBD therapy is under study. We previously presented the development of an invasive strain of *Lactococcus lactis* (*L. lactis*) producing Fibronectin Binding Protein A (FnBPA) which was capable of delivering, directly to host cells, a eukaryotic DNA expression vector coding for IL-10 of *Mus musculus* (pValac:*il-10*) and diminish inflammation in a trinitrobenzene sulfonic acid (TNBS)-induced mouse model of intestinal inflammation. As a new therapeutic strategy against IBD, the aim of this work was to evaluate the therapeutic effect of two *L. lactis* strains (the same invasive strain evaluated previously and the wild-type strain) carrying the therapeutic pValac:*il-10* plasmid in the prevention of inflammation in a dextran sodium sulphate (DSS)-induced mouse model.

**Results:**

Results obtained showed that not only delivery of the pValac:*il-10* plasmid by the invasive strain *L. lactis* MG1363 FnBPA+, but also by the wild-type strain *L. lactis* MG1363, was effective at diminishing intestinal inflammation (lower inflammation scores and higher IL-10 levels in the intestinal tissues, accompanied by decrease of IL-6) in the DSS-induced IBD mouse model.

**Conclusions:**

Administration of both *L. lactis* strains carrying the pValac:*il-10* plasmid was effective at diminishing inflammation in this murine model of experimental colitis, showing their potential for therapeutic intervention of IBD.

## Background

Inflammatory bowel diseases (IBD), including ulcerative colitis (UC) and Crohn’s disease (CD), are characterized by spontaneous and chronic inflammation of the gastrointestinal tract (GIT). Despite much research in the last years, the exact etiology and pathogenesis of these disorders remain unclear; however, it is nowadays generally accepted that IBD are caused by dysregulation of the mucosal immune system in relation to the native intestinal microbiota in genetically susceptible individuals [1]. Current treatments for IBD are restricted to the use of anti-inflammatory drugs, immunosuppressants and antibiotics, which although showing moderate therapeutic effect, present serious side effects and reveal that better, cheaper and longer lasting drugs are necessary [2].

Interleukin-10 (IL-10) is one of the most important anti-inflammatory cytokines involved in the intestinal immune system [3] and because of its immunosuppressive activity and its central role in downregulating inflammatory cascades [4] it presents itself as a good therapeutic candidate against IBD [5]. Recombinant human IL-10 raised hope when first used in the 90s in CD patients as the treatment led to remission in patients that were otherwise refractory to treatment [6]; however, two large, multi-centered follow-up studies using subcutaneous dosing were unable to confirm the results [7,8]. Moreover, systemic treatment with IL-10 showed to be quite limiting because of its short half-life (1.1-2.6 h) and requirement of high protein concentration (20 μg/kg), increasing the cost of production, discomfort and secondary effects in the patients [9]. On the other hand, oral treatment with IL-10 has also shown to be limited due to its extreme sensitivity to the environment of the GIT and therefore survival in it [10].

New approaches to yield more specific delivery of IL-10 to the intestinal mucosa and prevent the drawbacks associated to systemic and oral administration led to the development of IL-10-producing *Lactococcus lactis* (*L. lactis*) strains [11]. In 2003, a biological containment system for human IL-10-producing *L. lactis*[12] showed to be safe and improved the disease when tested in CD patients in phase I clinical trials [13]; however, clinical results did not reveal a statistically significant difference in mucosal healing between patients receiving the recombinant strains and placebo.

Since this strain produces IL-10 and releases it in the GIT, its clinical use for IBD is still hindered by IL-10’s sensitivity and poor survival in these hostile conditions. In this regard, a new eukaryotic DNA expression vector for delivery using lactococci, called pValac, which allows cloning of an ORF (Open Reading Frame) of interest, expression of the molecule by host cells, replication both in *E. coli* and in *L. lactis* and selection of bacteria, was firstly constructed in 2009 [14]. Its potential to deliver DNA and trigger DNA expression by epithelial cells has already been demonstrated *in vitro*[15] and *in vivo*[16]. This strategy does not only fulfil the aforementioned advantages, but also combines the advantages of mucosal immunity, safety of using non-pathogenic bacteria, technique simplicity and low cost of DNA therapy. Moreover, ingested *L. lactis* strains pose no risk to the individuals as these bacteria are quickly degraded and only around 20-30% reach the sites of inflammation, their transit through the gastrointestinal tract takes between 2 to 3 days and they are incapable of multiplying in the body or become part of the normal gut flora.

Our research group recently evaluated a recombinant invasive *L. lactis* strain expressing the *Staphylococcus aureus* Fibronectin Binding Protein A (FnBPA), harbouring the eukaryotic DNA expression vector pValac coding for the anti-inflammatory cytokine IL-10 of *Mus musculus* (*L. lactis* MG1363 FnBPA + pValac:*il-10*), for *in situ* expression of IL-10 and therefore higher, more efficient and direct production of this cytokine at the sites of inflammation. This strategy showed to be efficient at diminishing inflammation in a TNBS-induced inflammatory mouse model [17].

The aim of the present work was to evaluate and compare the therapeutic capacity of two *L. lactis* strains, the invasive *L. lactis* MG1363 FnBPA + strain and the wt *L. lactis* MG1363, both carrying the pValac:*il-10* plasmid, for the prevention of experimental IBD in a DSS-induced mouse model.

## Methods

### Bacterial strains, growth conditions and plasmid

The bacterial strains used in this work are listed in Table 1. *E. coli* TG1 was aerobically grown in Luria-Bertani (LB) medium at 37°C with vigorous shaking whereas all *L. lactis*[18] strains were grown in M17 medium (Difco, Sparks, MD, USA) supplemented with 0.5% glucose (GM17) at 30°C without shaking. Recombinant *E. coli* were selected by addition of 10 μg/mL chloramphenicol (Cm) while recombinant *L. lactis* were selected by addition of 10 μg/mL Cm and/or 5 μg/mL of erythromycin (Ery). For animal trials, *L. lactis* cultures grown until an OD_600_ of 1.0-1.2 were previously stocked in glycerol 80% (1:4) and on day of use they were centrifuged in order to eliminate any remaining traces of the antibiotic and medium and resuspended in 100 μL of saline solution (0.15 M NaCl) for animal feedings.

**Table 1:** Bacterial strains used in this work

**Bacterial Strain**	**Characteristics**	**Reference**
*Escherichia coli* (*E.coli*) TG1	[supE, hsd, ∆5, thi, ∆lac-proAB), F’(traD36 proAB-lacZ∆M15)]	Invitrogen (São Paulo, Brazil)
*Escherichia coli* (*E.coli*) TG1 (pValac:*il-10*)	(pCMV/Cm^R^/RepA/RepC/*IL-10*)	[17]
*Lactococcus lactis* (*L. lactis*) MG1363	*L. lactis* subsp. *cremoris*^b^	Laboratory of Cellular and Molecular Genetics (LGCM), Federal University of Minas Gerais (UFMG), Brazil
*Lactococcus lactis* (*L. lactis*) MG1363 pValac:*il-10*	*L. lactis* MG1363 strain carrying the pValac:*il-10* plasmid	This work
*Lactococcus lactis* (*L. lactis*) MG1363 FnBPA	*L. lactis* MG1363 strain expressing FnBPA of *S. aureus*	[19]
*Lactococcus lactis* (*L. lactis*) MG1363 FnBPA pValac:*il-10*	*L. lactis* MG1363 strain expressing FnBPA of *S. aureus* carrying the pValac:*il-10* plasmid	[17]

As previously shown by del Carmen and co-workers, the pValac:*il-10* plasmid harbours an eukaryotic region containing the CytoMegaloVirus promoter (pCMV), the IL-10 ORF of *Mus musculus* and the polyadenylation signal of bovine growth hormone (BGH polyA), required for gene expression by eukaryotic host cells, as well as a prokaryotic region containing the RepA/RepC replication origins for both *E. coli* and *L. lactis*, respectively, and a Cm resistance gene for bacterial selection [14].

### DNA manipulations

General DNA manipulation techniques were carried out according to standard procedures. Unless otherwise indicated, DNA restriction and modification enzymes were used as recommended by the suppliers. DNA fragments were isolated from agarose gels using the Illustra GFX PCR DNA and Gel Band Purification Kit (GE Healthcare Life Sciences, Chalfont St. Giles, UK). PCR amplifications were performed using AccuPrimePfx DNA Polymerase (Invitrogen, Carlsbad, CA, USA) and/or GoTaq DNA Polymerase (Invitrogen, Carlsbad, CA, USA) in a DNA thermocycler (MJ Research, Inc., Minnesota, USA). Plasmid DNA from *E. coli* and *L. lactis* was isolated as previously described [19] with the following modifications: for plasmid DNA extraction from *L. lactis*, the first step included addition of TES (25% sucrose, 1 mM EDTA, 50 mMTris-HCl, pH 8) containing lysozyme (10 mg/mL) for 30 min at 37°C to prepare protoplasts. Electroporation of *E. coli* and *L. lactis* was performed as previously described [20]. *E. coli* transformants were plated on LB agar plates containing the required antibiotic for 24 h at 37°C, whereas *L. lactis* transformants were plated on GM17 agar plates containing the required antibiotic and were counted after 2-days incubation at 30°C.

### Induction of intestinal inflammation and feeding procedure in mice

Conventional female C57BL/6 mice [21] weighing approximately 18 g, obtained from the inbred closed colony (CEBIO) maintained at the Federal University of Minas Gerais (UFMG – Belo Horizonte, Brazil), were used to evaluate the therapeutic effect of *L. lactis* MG1363 pValac:*il-10* and *L. lactis* MG1363 FnBPA + pValac:*il-10* in the prevention of intestinal inflammation in a DSS-induced mouse model. Procedures and manipulation of animals followed the rules of the ethics and research committees of the Biological Institute of the UFMG and all animal protocols were approved by the Ethics Committee on Animal Experiments (CETEA). All animals were maintained in collective cages (6 animals/cage) in an environmentally controlled room with a 12-hour light/dark cycle and unlimited free access to water (or DSS solution) and food. After acclimatization for 17 days, colonic inflammation was induced by the addition of 1.5% (w/v) DSS (MW 40,000-50,000; USB Affymetrix, Santa Clara, CA, USA) in drinking water for 7 consecutive days. Liquid consumption was monitored to ensure that all mouse groups consumed similar volumes (4 ± 2 mL) of the DSS solution daily. For experimental procedure, mice were divided in 6 experimental groups: i) control group which received 100 μl NaCl intragastrically while no DSS was added to the drinking water; ii) DSS group which received 100 μl NaCl intragastrically while DSS was added to the drinking water and iii) *L. lactis* MG1363 group (wt group), iv) *L. lactis* MG1363 pValac:*IL-10* group, v) *L. lactis* MG1363 FnBPA + group and vi) *L. lactis* MG1363 FnBPA + pValac:*il-10* group, which all received intragastrically 100 μl of the corresponding bacterial strain as suspension, at a dose of 2×10^9^ CFU/100 μl, in NaCl. The intragastric administration of NaCl or the bacterial suspensions was once daily since the day before the beginning of DSS administration until sacrifice. At day 9, mice were sacrificed by cervical dislocation for organ collection.

### Macroscopic and microscopic (histological) assessment of intestinal inflammation

On the day of sacrifice (day 9), colons were removed, excised and visually inspected for macroscopic evaluation to assess colonic inflammation [disease activity index (DAI)], using a scoring system in which the following features were graded: body weight loss (0, no loss; 1, 1-5% loss; 2, 5-10% loss; 3, 11-15% loss and 4, >15% loss), diarrhoea (0, absent; 2, moderate; 4, severe) and rectal bleeding (0, absent; 2, moderate; 4, severe). The macroscopic damage score was calculated from the score of all clinical signs with a maximum score of 12. For histological inflammation scoring [histological activity index (HAI)], samples of the colon were fixed in 10% formalin in phosphate-buffered saline (PBS), embedded in paraffin, cut into 3–5-μm sections and stained with haematoxylin–eosin (H&E-staining) for microscopic analysis. These sections were blindly scored based on a semi quantitative scoring system previously described [22] in which the following features were graded: extent of destruction of the mucosa’s architecture (0, normal; 1, 2 and 3, light, moderate and extensive damage, respectively), presence and degree of cellular infiltration (0, normal; 1, 2 and 3, light, moderate and transmural infiltration, respectively), extent of muscle thickening (0, normal; 1, 2 and 3, light, moderate and extensive thickening, respectively), presence or absence of crypt abscesses (0, absent and 1, present) and the presence or absence of goblet cell depletion (0, absent and 1, present). The histological damage score was calculated as the addition of the scores corresponding to each feature. High macroscopic and histological damage scores indicate increased damage in the intestines.

### Secretory IgA (sIgA) assay

Levels of sIgA in the intestinal fluid were determined by enzyme linked immunosorbent assay (ELISA) as previously described [23]. Briefly, microtitre plates (NUNC, Thermo Scientific, Waltham, MA, USA) were coated with goat anti-mouse UNLB antibody (Southern Biotechnology, Birmingham, AL, USA) in coating buffer (pH 9.8) overnight at 4°C. Wells were then washed with a saline 0.05% tween solution and blocked with 200 μL of PBS with 0.05% (w/v) casein for 1 h at room temperature. The supernatants obtained from the intestinal fluids after centrifugation at 432 *g* for 20 min at 4°C and the diluted standards in PBS-0.25% casein (1:10) were then added to the plate and incubated for 1 hour at 37°C. After washing, goat anti-mouse IgA HRP (Southern Biotechnology, Birmingham, AL, USA) was added and plates were incubated for 1 h at 37°C. The colour reaction was developed at room temperature with the addition of 100 μL/well of orthophenylenediamine (OPD) (1 mg/mL) (Sigma, St. Louis, MO, USA), 0.04% H_2_O_2_ substrate in sodium citrate buffer. The reaction was finally stopped by the addition of 20 μL/well of 2 N H_2_SO_4_. Absorbance was measured at 492 nm using a Bio-Rad Model 450 Microplate Reader. Results were expressed as concentration (μg/mL), according to the standard curve.

### Colon tissue preparation and cytokine assays

For cytokine assays, colons were weighed and homogenized in PBS containing 0.05% (v/v) Tween-20, 0.1 mM phenylmethylsulphonyl fluoride, 0.1 mM benzethonium chloride, 10 mM EDTA and 20 KIU AprotininA using a tissue homogenizer (1 mL/0.1 g). Suspensions were centrifuged at 600 *g* for 10 min at 4°C and the supernatants collected for cytokine assay. Concentrations of IL-10, TNF-α, IL-6, IL-17 were measured by ELISA as described previously [23]. Briefly, after coating microtitre plates (NUNC, Thermo Scientific, Waltham, MA, USA) with purified monoclonal antibodies reactive to mouse cytokines IL-10, TNF-α, IL-6, IL-17 (BD, New Jersey, USA), standards and samples were added and incubated overnight at 4°C. Biotinylated monoclonal antibodies anti-mouse IL-10, TNF-α, IL-6, IL-17 were added and incubated for 1 h at room temperature, after which peroxidase-labelled streptavidin (Sigma, St. Louis, MO, USA) was added. A colour reaction was developed at room temperature with 100 μL/well of OPD (1 mg/mL) and 0.04% H_2_O_2_ substrate in sodium citrate buffer. The reaction was stopped by the addition of 20 μL/well of 2 N H_2_SO_4_. Absorbance was measured at 492 nm using a Bio-Rad Model 450 Microplate Reader. Results were expressed as concentration of each cytokine (pg/mL), according to the respective standard curve.

### Statistical analysis

Statistical analyses were performed using the GraphPad Prism 5.0 software (San Diego, CA, USA) and all results were expressed as mean ± standard deviation (SD). Significance of differences among groups was assessed by Student’s t-test or analysis of variance (ANOVA) followed by a Tukey comparison post-hoc test. Means were considered statistically different when p < 0.05.

## Results

### Construction of *L. lactis* MG1363 pValac:*il-10*

The pValac:*il-10* plasmid was previously constructed by del Carmen and co-workers and used to construct the invasive *L. lactis* MG1363 FnBPA + pValac:*il-10* strain. This strain was tested in an experimental IBD mouse model induced by TNBS and showed to diminish inflammation and damage scores [17]. In this work, we constructed the non-invasive *L. lactis* MG1363 pValac:*il-10* strain; the construction was confirmed by PCR (data not shown). This strain was tested together with the invasive strain in the experimental IBD mouse model induced by DSS.

### *L. lactis* strains carrying the pValac:*il-10* plasmid show anti-inflammatory properties in the DSS-induced colitis in mice

DSS is a well-established animal model of mucosal inflammation that has been used for over two decades in the study of IBD pathogenesis and preclinical studies [8] and when administrated in drinking water induces an acute inflammation. C57BL/6 mice were used for the DSS study as these animals are highly susceptible to DSS-induced colitis and do not naturally recover from this disease [21].

**Figure 1:**
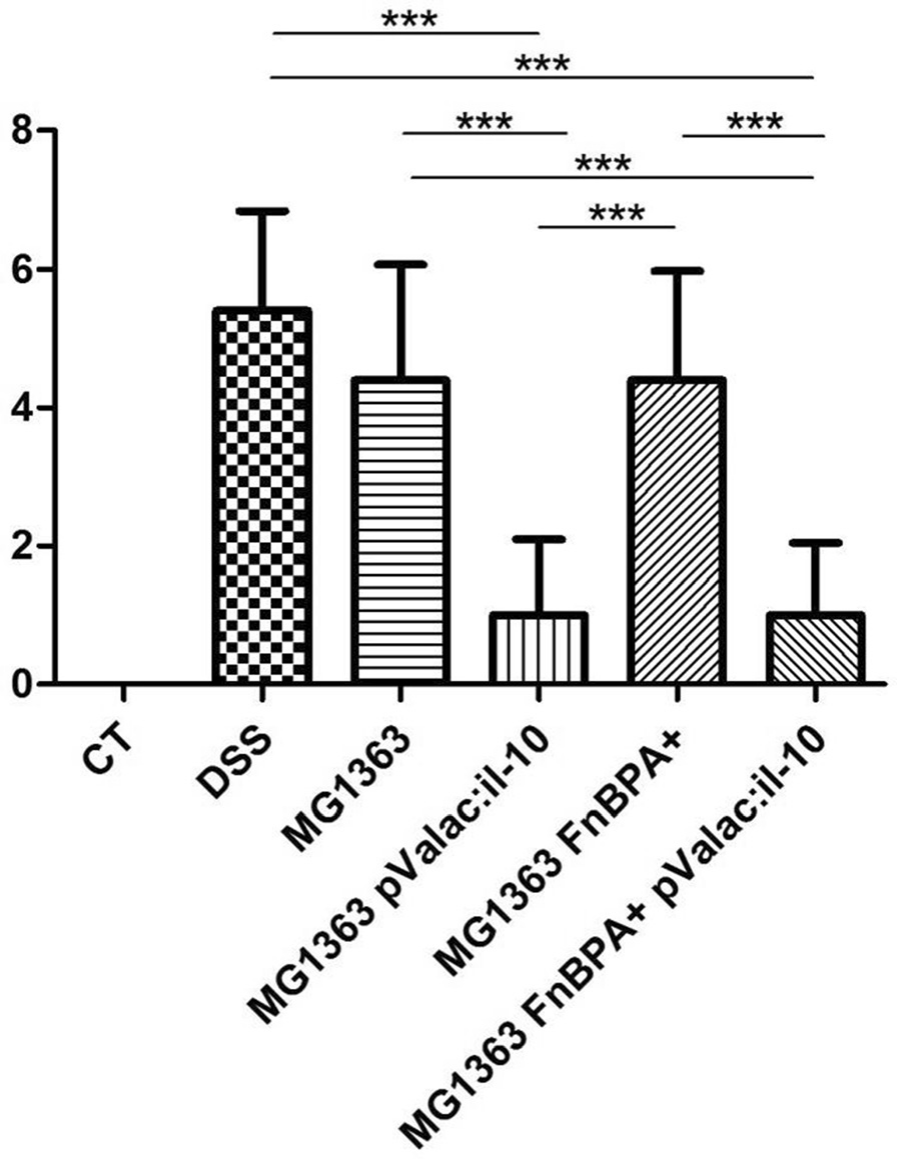
**Inflammatory macroscopic score of experimental colitis.** Macroscopic score of mice treated with DSS that received the different *L. lactis* strains compared to the DSS group without any specific administration. Disease activity index (DAI) was scored for body weight loss, diarrhoea and rectal bleeding. Bars represent the mean ± SD of 6 mice per group of two independent experiments. Asterisks represent statistical significance (**p < 0*.*01 or ***p < 0*.*0001). All groups are statistically significant to the control group (***p < 0.0001).

**Figure 2:**
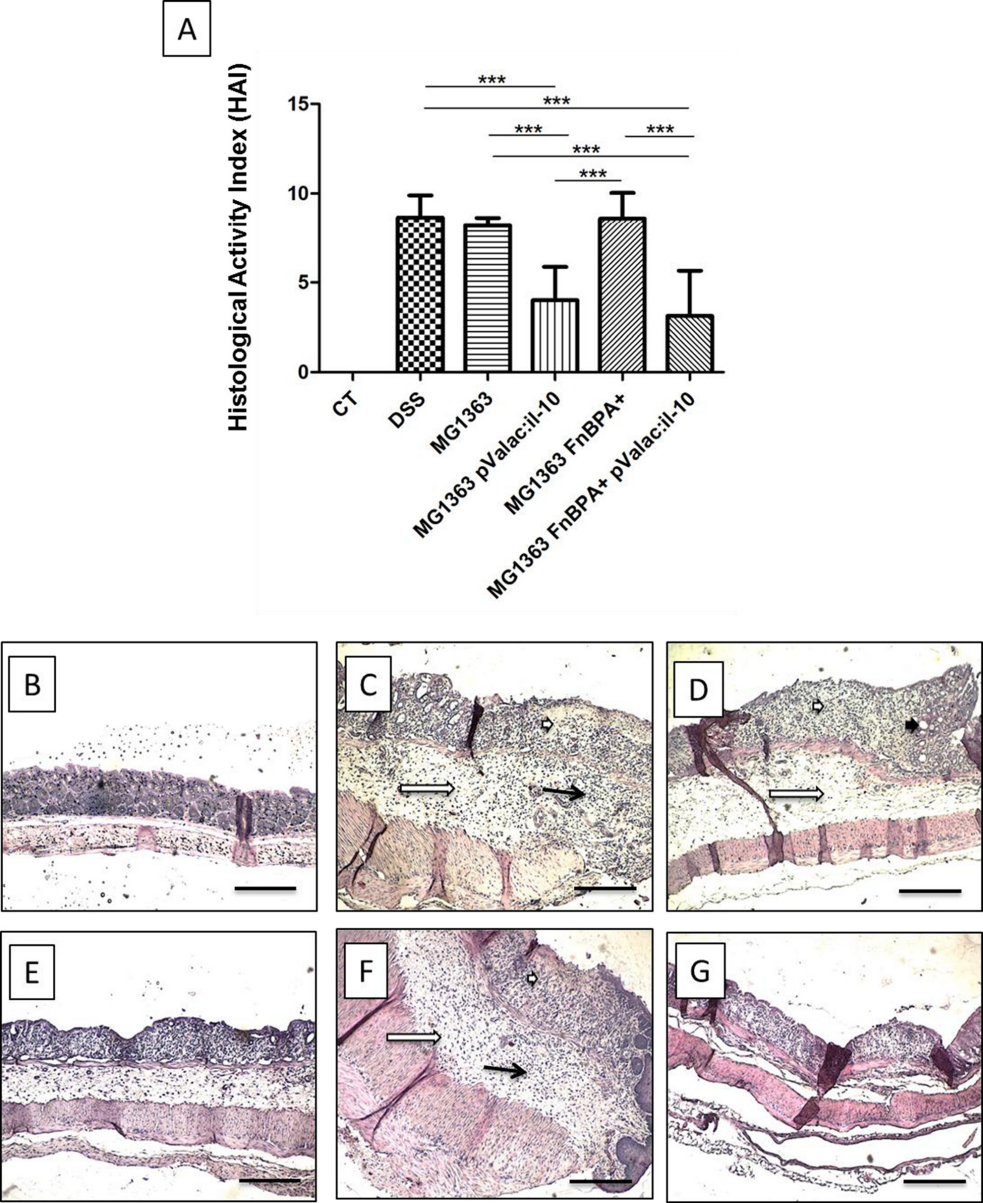
**Inflammatory microscopic score of experimental colitis and histopathology of intestinal changes between all groups.** Macroscopic score of mice treated with DSS that received the different *L. lactis* strains compared to the DSS group without any specific administration **(A)**. Histological activity index (HAI) was obtained from the colonic tissues stained by H&E. Bars represent the mean ± SD of 6 mice per group of two independent experiments. Asterisks represent statistical significance (**p < 0.01 or ***p < 0.0001). All groups are statistically significant to the control group (***p < 0.0001). Representative photos obtained from the proximal colon of a mouse from control group: Control group **(B)**, DSS group **(C)**, and the groups of mice treated with DSS that received *L. lactis* MG1363 **(D)***L. lactis* MG1363 pValac:*il-10***(E)** Proximal colon of diseased mice that received the *L. lactis* MG1363 FnBPA + **(F)** or *L. lactis* MG1363 FnBPA + pValac:*il-10***(G)**. The bar on each image represents 100 μm. The short white arrows show the depletion of goblet cells; the long white arrow, intense oedema in the submucosa; the short black arrow, crypt abscesses; and the long black arrow, inflammatory infiltrate (especially of macrophages).

Similar to the results previously obtained in a TNBS-induced colitis mouse model, *L. lactis* MG1363 FnBPA + pValac:*il-10* was also capable of diminishing inflammation in the DSS-induced colitis mouse model (Figures 1 and 2). It was also observed that the non-invasive variant, *L. lactis* MG1363 pValac:*il-10*, was also capable of decreasing the inflammation in this model. Overall, both groups that received the *L. lactis* strains carrying the pValac:*il-10* (pValac:*il-10* groups) showed a lower DAI (Figure 1) and therefore significant decrease of inflammation, when compared to the DSS control group and the groups receiving *L. lactis* MG1363 and *L. lactis* MG1363 FnBPA+, which presented more diarrhoea and bleeding, which was almost absent in animals from the pValac:*il-10* groups. The colon length of each animal was also measured as a marker of inflammation-caused injury. As expected, there was a significant shortening of the colon induced by DSS treatment (6.92 cm), and the administration of *L. lactis* MG1363 (6.96 cm) or *L. Lactis* MG1363 FnBPA + (6.93 cm) did not improve it. However, the pValac:*il-10* groups showed a significant improvement in colon length, *L. lactis* MG1363 pValac:*il-10* (7.97 cm) and *L. lactis* MG1363 FnBPA pValac:*il-10* (8.01 cm), when compared to the other groups induced with DSS, resembling more the colon lengths of the control group (8.16 cm). Regarding body weight loss, all groups maintained similar weight during the 8 days of experiment (data not shown).

To evaluate the colons microscopically, a HAI was performed for all groups, and showed that animals from the pValac:*il-10* groups presented a significant decrease of the damage score when compared to the DSS group and the mice that received *L. lactis* MG1363 or *L. lactis* MG1363 FnBPA + (Figure 2A).

Histologically, all samples from the colon of healthy control mice (group without DSS treatment) presented themselves within normal standards. The architecture integrity of the colon was maintained, with goblet cells, thin submucosa without signs of congestion or oedema and absence of inflammatory infiltrate in the mucosa and submucosa (Figure 2B). On the other hand, the colitis control group (DSS group, Figure 2C) presented intense alteration in the mucosal architecture of their colons, with areas of intense ulceration, absence of glands in large parts of the mucosa and accentuated depletion of goblet cells. It was also possible to observe crypt abscesses and the submucosa was marked by an intense oedema and inflammatory infiltrate constituted principally by macrophages. Moreover, thickening of the muscle layer was also observed. No significant differences were observed when animals received *L. lactis* MG1363 (Figure 2D) or *L. lactis* MG1363 FnBPA + (Figure 2F) strains, compared to the DSS treated control. However, groups that received *L. lactis* MG1363 pValac:*il-10* (Figure 2E) or *L. lactis* MG1363 FnBPA + pValac:*il-10* (Figure 2G) presented a milder pathological picture, with tendency to normality, a smaller compromised area and a reduction in the intensity of the lesion. Reduced inflammatory infiltrates without erosion of the epithelium, light oedema and diminished depletion of goblet cells, partially preserving the mucosal architecture, were also observed.

### The pValac:*il-10* plasmid increases the production of secretory IgA

**Figure 3:**
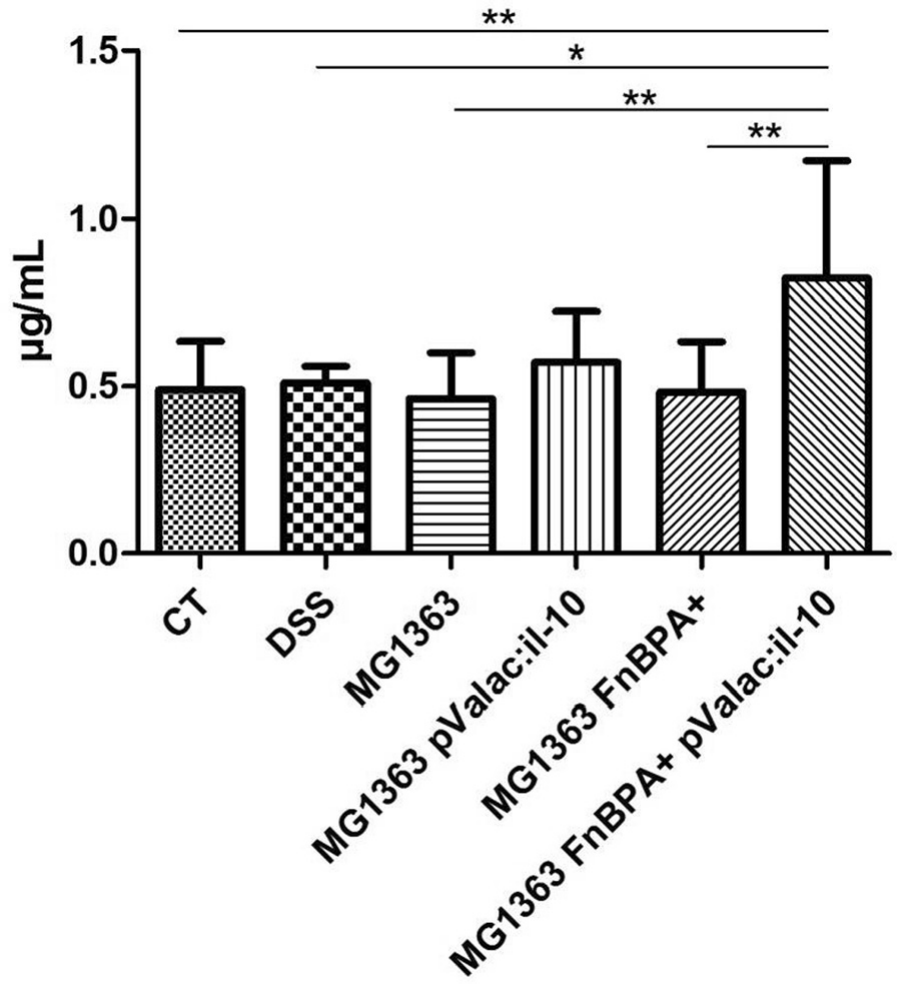
**Influence of DSS and different bacterial administrations on gut secretory IgA.** Intestinal fluid was collected and total sIgA was measured by ELISA of wild-type mice, DSS wild-type mice and diseased mice that received the *L. lactis* MG1363 strain, *L. lactis* MG1363 pValac:*il-10* strain, *L. lactis* MG1363 FnBPA + strain or *L. lactis* MG1363 FnBPA + pValac:*il-10* strain during treatment. Bars represent the mean N = 6 ± SD of two independent experiments. Asterisks represent statistical significance *p < 0.05 or **p < 0.001).

Secretory IgA creates a first-line of defence against mucosal compromise that is lost during IBD. Therefore, sIgA levels from intestinal fluids of mice were also analysed. It was observed that only mice that received the *L. lactis* MG1363 FnBPA + pValac:*il-10* strain showed a significant increase in the concentration of sIgA when compared to those from the control group, DSS group and the groups that did not receive the strains carrying the pValac:*il-10* plasmid (Figure 3). Moreover, this strain also induced an increased sIgA production when compared to the non-invasive *L. lactis* MG1363 pValac:*il-10* strain.

### The administration of *L. lactis* carrying the pValac:*il-10* plasmid modulates the production of cytokines in the intestinal tissues

**Figure 4:**
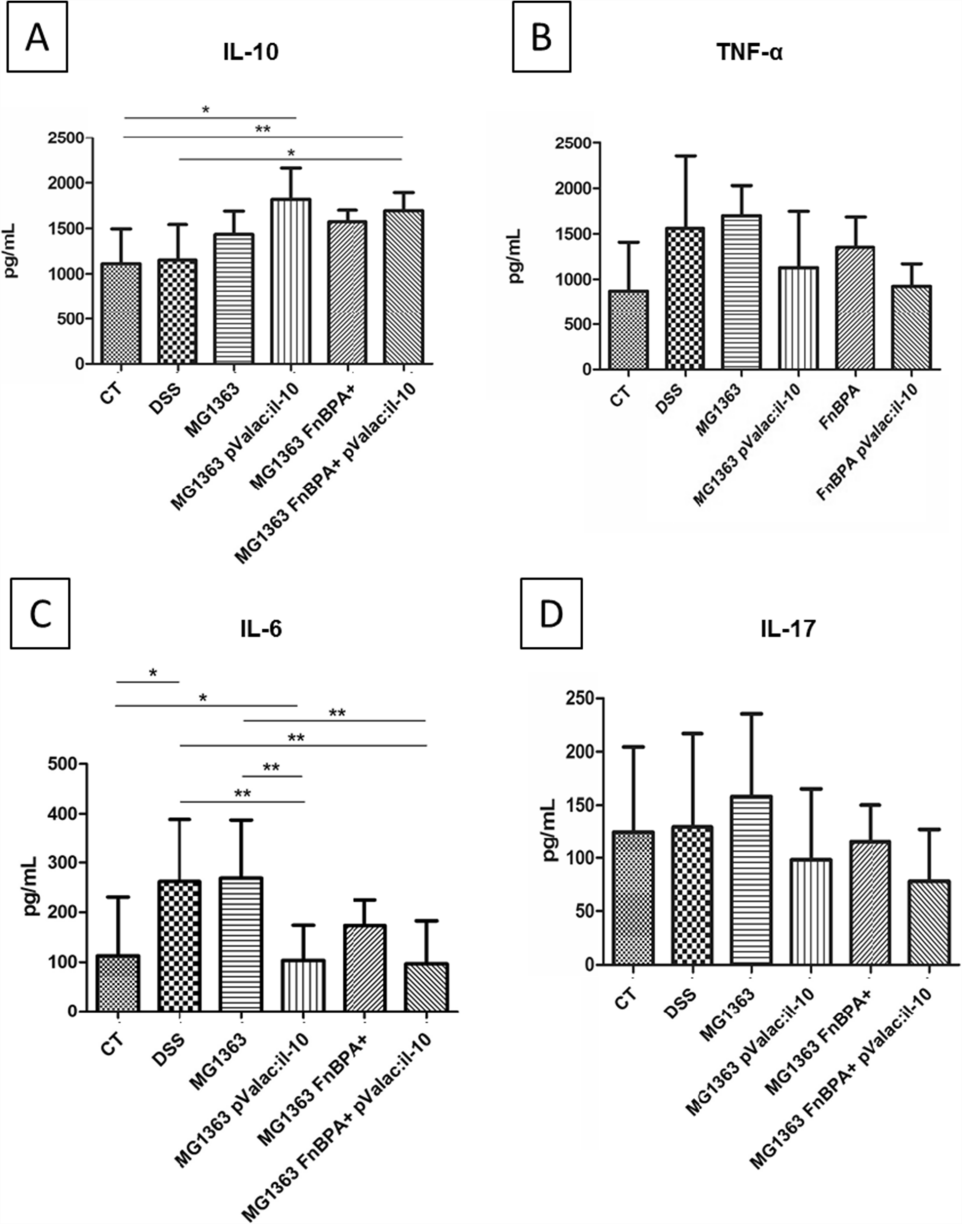
**The pValac:*IL-10 *plasmid modulates the production of cytokines in intestinal tissues.** IL-10 **(A)** TNF-α **(B)**, IL-6 **(C)** and IL-17 **(D)** levels in the intestinal tissues of mice from control group (CT), DSS group and mice that received the *L. lactis* MG1363, *L. lactis* MG1363 pValac:*il-10*, *L. lactis* MG1363 FnBPA + or *L. lactis* MG1363 FnBPA + pValac:*il-10*. Bars represent the mean ± SD of 6 mice per group of two independent experiments. Asterisks represent statistical significance (*p *<* 0*.*05 or **p *<* 0*.*001).

Administration of DSS to mice leads to a macrophage-induced inflammation and tissue damage accompanied by a cellular cytotoxic-mediated inflammatory response (macrophage/T_h_1/T_h_17 chemotactic profile) during the progression of colitis [24]. In this regard, in order to determine if the pValac:*il-10* plasmid could modulate the cytokine level in diseased animal, IL-10, TNF-α, IL-6, IL-17 were measured from colonic tissues. Mice from the *L. lactis* MG1363 pValac:*il-10* group showed significantly higher IL-10 levels compared with the control group, while animals from the *L. lactis* MG1363 FnBPA + pValac:*il-10* group showed higher IL-10 levels than the animals from the control and DSS groups (Figure 4A). Regarding pro-inflammatory cytokines, TNF-α (Figure 4B) and IL-17 (Figure 4D) levels did not show significant differences between the control and test groups, while IL-6 levels showed significant differences between some of the test and control groups (Figure 4C). IL-6 increased significantly (*p < 0.05) in the DSS group when compared to the control group, and no significant differences with the groups that received *L. lactis* MG1363 or *L. lactis* MG1363 FnBPA + were observed. The levels of IL-6 were significantly lower (**p < 0.001) in the pValac:*il-10* groups compared to the DSS group, resembling the values of the control group.

## Discussion

Interleukin 10’s interest in the attempt of developing an efficient therapy against IBD has grown since it was observed that IL-10 knockout (IL-10^−/−^) mice develop spontaneous enterocolitis when not maintained in germ-free conditions [25]. Although many advances have been made there still does not exist any treatment for IBD, showing that the major drawback to use IL-10 is its correct administration and directioning to the sites of inflammation.

In order to overcome these problems, new strategies based on local delivery of IL-10 to the intestinal mucosa have been developed; high IL-10 concentration at these sites would outweigh the need for high doses and as such avoid the increase of pro-inflammatory cytokine production and other undesired side effects.

One such strategy consists in the use of polymer-based microparticles. These particles encapsulate gelatine nanoparticles containing plasmid DNA expressing murine IL-10 and are capable of releasing these nanoparticles directly at the desired site of action. These IL-10 gene containing particles have shown to successfully reduce the levels of inflammatory cytokines as well as disease activity scores in the TNBS-induced model of colitis [26]. However, despite promising, scale-up to an industrial level appears to be very expensive and complicated.

Another strategy by Yao and collaborators consists in a *Bifidobacterium longum* strain capable of secreting human IL-10 which was capable of alleviating inflammatory damage of colonic tissue in a DSS-induced mouse model by blocking the colitis-activated NF-κB pathways and upregulating CD4 + CD25 + Foxp3+ T_reg_ in blood and mesenteric lymph nodes [27].

As such, in order to improve IL-10 delivery to the sites of inflammation using *L. lactis*, a *L. lactis* strain expressing FnBPA [15,28], that has the capacity to efficiently internalize and trigger recombinant DNA expression by human epithelial cells, was used to deliver a eukaryotic expression vector coding for IL-10 of *Mus musculus*, pValac:*il-10*, directly to host cells in the GIT for recombinant *in situ* IL-10 production. This new strategy recently showed to be capable of diminishing inflammation in a TNBS-induced mouse model [17].

Following these results and in order to evaluate the therapeutic effect of the pValac:*il-10* plasmid in a larger context, in the present work we explored the potential of this therapeutic plasmid in a DSS-induced mouse model delivered by two *L. Lactis* strains as a new therapeutic strategy for the prevention of intestinal inflammation. *L. lactis* MG1363 FnBPA + expresses FnBPA, which confers the strain the capacity to mediate adhesion to host tissue and bacterial uptake by eukaryotic cells. To evaluate if this characteristic results in improved anti-inflammatory effect, it was compared with the non-invasive *L. lactis* MG1363 strain. Our results showed that both strains were capable of delivering the eukaryotic expression vector to host cells directly at the sites of inflammation leading to *in situ* IL-10 production, avoiding strong and undesired side-effects, and diminishing the severity of inflammation by maintaining an anti-inflammatory environment in the gut.

DSS has the intrinsic capacity to disrupt the epithelial cell barrier, causing normal gut substances to activate mucosal macrophages, which in turn produce immunomodulatory cytokines. It is generally accepted that DSS is directly toxic to gut epithelial cells of the basal crypts and affects the integrity of the mucosal barrier [29]. The DSS concentration used in the present model triggered an acute colonic inflammation accompanied by diarrhoea and rectal bleeding and did not lead to mortality. The administration of strains carrying the pValac:*il-10* plasmid statistically lowered the macroscopic score compared to the DSS group, regarding diarrhoea and rectal bleeding. No weight loss was observed within the different groups, but this has already been reported earlier [30]. At histological level, administration of strains carrying the pValac:*il-10* plasmid were capable of decreasing the severity of inflammation, with tendency to normality, demonstrating the anti-inflammatory effects of this strategy.

IgA is the most abundant immunoglobulin produced, in its secretory form, at mucosal sites. In the luminal mucous layer, sIgA protects the intestinal epithelium against colonization and invasion by pathogens or commensals [31] and therefore helps modulating and controlling inflammation. In our model, no significant decrease of sIgA was associated with the inflammation observed in mice treated with DSS that did not receive the pValac:*il-10*. The administration of bacteria carrying the pValac:*il-10* plasmid was accompanied by significantly increased levels of sIgA only in the intestinal fluid of mice that received the *L. Lactis* MG1363 FnBPA + pValac:*il-10* strain, compared to the DSS treated mice. Moreover, this strain also induced an increased sIgA production when compared to the *L. lactis* MG1363 pValac:*il-10* strain. We believe that the difference of IgA production induced by these two strains is due to the different cellular entry mechanism these bacteria use after oral administration. In this regard, *L. lactis* MG1363 FnBPA + pValac:*il-10* strain induced increased IgA production because these bacteria invade enterocytes in the epithelium, while *L. lactis* MG1363 pValac:*il-10* are captured by macrophages (as these bacteria have no invasion capacity), which does not directly lead to the induction of IgA. These results showed that the *L. Lactis* MG1363 FnBPA + pValac:*il-10* strain could not only have an anti-inflammatory effect in this model, but also promote the gut immunological barrier by limiting the penetration of bacteria into host tissues and therefore protect mice by helping to modulate inflammation. The expression of FnBPA by this strain might allow for a higher binding capacity and internalization by eukaryotic cells, thus enhancing the production of IL-10 and its anti-inflammatory properties.

Cytokines produced in the gut mucosa greatly influence the resulting immunological outcome; production of anti-inflammatory cytokines induces mucosal tolerance, while high levels of pro-inflammatory cytokines induce inflammation. Since cytokines are major mediators of inflammation and regulatory activity in the gut mucosa, we analysed the ability of *L. lactis* strains carrying the pValac:*il-10* plasmid to modulate the production of cytokines in the colonic tissues of mice. Mice that received the pValac:*il-10* plasmid, whether delivered by *L. lactis* MG1363 or *L. lactis* MG1363 FnBPA + (pValac:*il-10* groups), showed significantly higher IL-10 levels than the control group (healthy mice), probably due to a constant and higher production of IL-10 by the eukaryotic cells of these animals. It is also important to note that the expression of FnBPA by the bacterial strain carrying the pValac:*il-10* plasmid was associated to significantly higher IL-10 levels in the intestinal tissues compared to the DSS group, where this cytokine was produced as normal immune response against inflammation. However, these levels of IL-10 were not significantly different from those obtained in the *L. lactis* MG1363 pValac:*il-10* group, nor were associated with significant differences in the levels of the other cytokines tested in both pValac:*il-10* groups. The lack of significant difference between both strains carrying the pValac:*il-10* plasmid could be explained by the fact that both cellular entry mechanisms used by these bacteria (enterocytes invasion by the invasive strain and capture by macrophages by the non-invasive strains) lead to efficient and similar IL-10 production; however, by different cell types.

Pro-inflammatory cytokines, including TNF-α, IL-6 and IL-17, can be produced by T and B lymphocytes, macrophages and/or neutrophils, which are massively infiltrated in inflammatory lesions in mice with DSS-induced acute colitis. This DSS colitis switches from a T_h_1-T_h_17-mediated acute inflammation with increased levels of TNF-α, IL-6 and IL-17, to a predominant T_h_2-mediated inflammatory response that shows a decrease in TNF-α, IL-6 and IL-17 while increasing levels of anti-inflammatory cytokines IL-4 and IL-10 [24]. In our experimental acute model, at the time of sacrifice, the results only showed higher IL-6 levels in the DSS group and in the group of mice treated with DSS that received the *L. lactis* MG1363 strain, when compared to the control group (healthy mice). Significant decrease of this cytokine was observed in the intestinal tissues from mice that received the pValac:*il-10* plasmid, compared to the DSS group. These results were associated to the increased IL-10 levels obtained in those animals and confirm the anti-inflammatory capacity of both strains carrying the pValac:*il-10* plasmid at modulating the gut immune response.

## Conclusions

The results obtained in the present work confirm and strengthen our previous results that *L. lactis* MG1363 FnBPA + pValac:*il-10* shows to be a good candidate to maintain an anti-inflammatory status in the GIT and diminishing intestinal inflammation. Moreover, we here also showed that not only delivery of the pValac:*il-10* plasmid by the invasive strain *L. lactis* MG1363 FnBPA+, but also by the non-invasive *L. lactis* MG1363 strain, was effective at diminishing intestinal inflammation, showing that this strategy presents potential for therapeutic intervention of IBD. FnBPA expression was not related to more anti-inflammatory capacity; however, it was associated with significantly higher IL-10 levels in the intestinal tissues when compared to animals from the DSS group. Moreover, the highest levels of sIgA in the intestinal fluid where observed in the animals from the *L. lactis* MG1363 FnBPA + pValac:*il-10* group, showing better immunomodulatory effect by the invasive strain.

## Competing interests

The authors declare that they have no competing interests.

## Authors’ contributions

MZT designed the study, carried out the experiments and wrote the paper. SC and ACGS participated with the realization of the experiments and contributed to the scientific discussion. DCCM carried out all histological analyses and contributed with the scientific discussion. SL, AMLB, VA and JGLB helped to draft the manuscript and gave final approval for publication. JMC and AM conceived the study, participated in its design and coordination, helped to draft the manuscript and gave final approval for publication. All authors read and approved the final manuscript.

## Authors’ information

Jean G LeBlanc and Anderson Miyoshi Share credit in this work for senior authorship.
